# Realising early recognition of arthritis in times of increased telemedicine: the value of patient-reported swollen joints

**DOI:** 10.1136/annrheumdis-2020-219513

**Published:** 2021-01-07

**Authors:** Cleo Rogier, Bastiaan T van Dijk, Elisabeth Brouwer, Pascal H P de Jong, Annette H M van der Helm-van Mil

**Affiliations:** 1 Department of Rheumatology, Erasmus Medical Center, Rotterdam, Zuid-Holland, The Netherlands; 2 Department of Rheumatology, Leiden University Medical Center, Leiden, Zuid-Holland, The Netherlands; 3 Department of Rheumatology and Clinical Immunology, UMCG, Groningen, The Netherlands

**Keywords:** arthritis, rheumatoid, COVID-19, outcome assessment, health care, health services research

Early diagnosis and management of patients with inflammatory arthritis (IA) are critical to improve long-term patient outcomes. Assessment of joint swelling at joint examination is the reference of IA identification; early access clinics are constructed to promote this early recognition. Due to the COVID-19 pandemic, the face-to-face capacity of such services is severely reduced.^1^ This raises the concern of a major step backward after the important progress that has been made in the past 15 years.^1^ Telemedicine has recently become rapidly implemented. Although probably a valuable alternative in the management of established rheumatoid arthritis (RA), there is also the fear that this might cause delay in the speed of diagnosis.^2^ A symptom that evidently raises suspicion for IA during remote evaluation is the presence of patient-reported swelling. This symptom is also included in triage tools.^3 4^

The accuracy of patient-reported swelling in comparison with joint examination has been extensively evaluated in established RA. Heterogeneous results are reported; correlation coefficients were higher when patient scored their swelling on mannequins (ρ: 0.31–0.67) than when determined with questions.^5^ Hypothetically, the accuracy of patient-reported joint swelling for first recognition of IA is different than for flare detection in patients with established RA. To promote evidence-based care in the era of telemedicine, we determined the accuracy of patient-reported joint swelling for actual presence of IA in persons suspected of IA by general practitioners (GPs).

Data from two Dutch Early Arthritis Recognition Clinics were studied. These are screening clinics (1.5 lines setting) where GPs send patients in case of doubt on IA. At this clinic, patients were asked to mark the presence of swollen joints on a mannequin with 52 joints (42 joints were used for this analysis, see online supplemental text/figure S1). Subsequently, an experienced rheumatologist performed joint examination (see online supplemental text). Clinically apparent IA of ≥1 joint was the reference to calculate sensitivity, specificity, positive and negative likelihood ratios (LR+ and LR−) and positive and negative predictive value (PPV and NPV) on patient level. Pearson correlation coefficients (ρ) were determined. Predictive values depend on the prevalence of a disease in a population. Because the prevalence of IA in a 1.5 lines setting will differ from a primary care setting, post-test probabilities of IA were estimated for two lower prior-test probabilities as example, namely 20% (estimated probability in patients GPs believe IA is likely) and 2% (prior-test probability with less preselection by GPs), using likelihood ratios and nomograms (online supplemental figures S2 and S3).

10.1136/annrheumdis-2020-219513.supp1 Supplementary data

A total of 1637 consecutive patients were studied. Patient characteristics are presented supplementary (online supplemental table S1). Median symptom duration was 13 weeks. Seventy-six per cent of patients marked ≥1 swollen joint at the mannequin. Forty-one per cent of patients had ≥1 swollen joint at examination by rheumatologists. ρ was 0.20 (patient level) to 0.26 (joint level).

**Figure 1:**
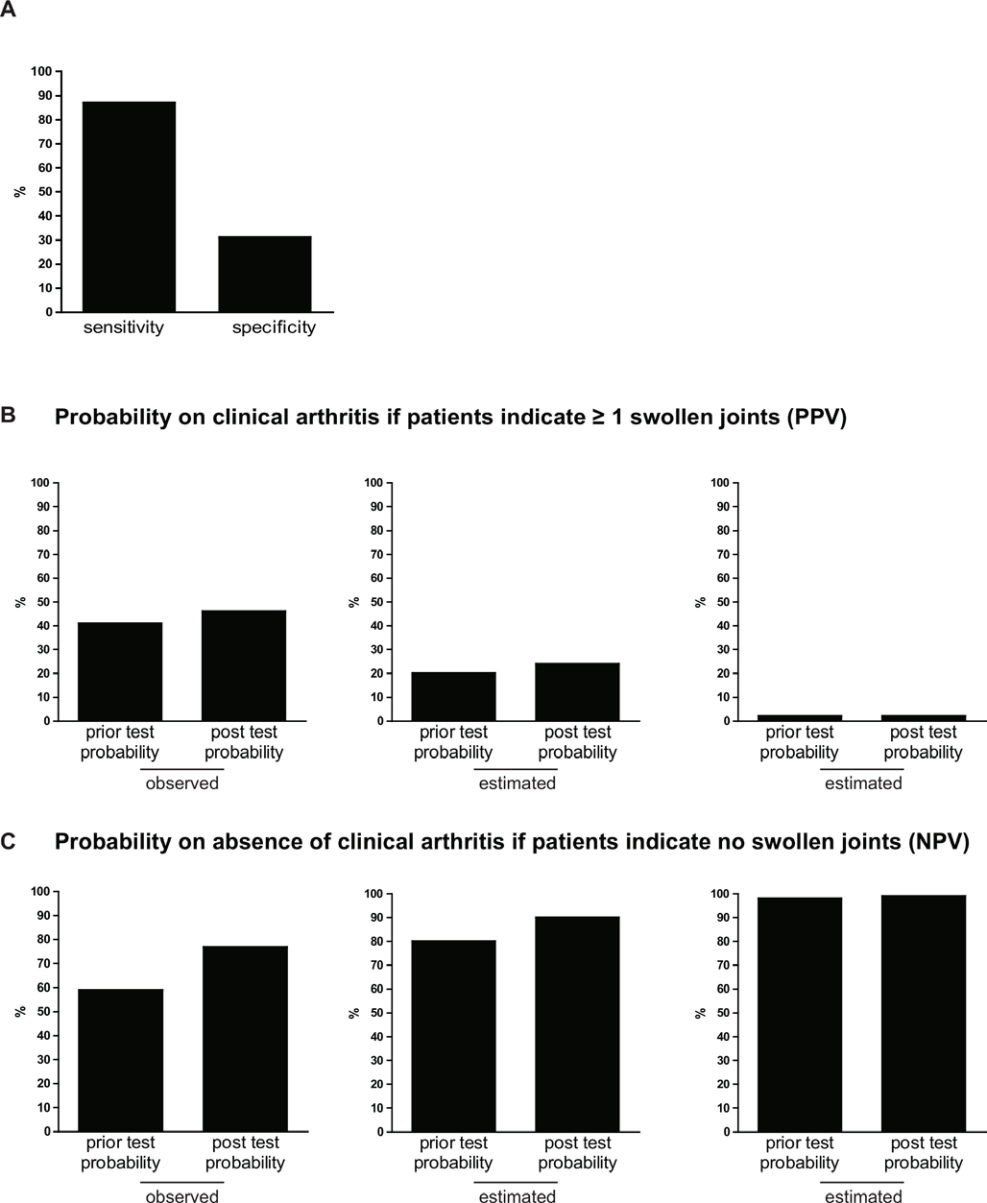
Test characteristics of patient-reported joint swelling (A) and predictive values (B and C), demonstrating the limited value of patient-reported joint swelling for detection of IA in three settings with different prior probabilities. (A) Sensitivity and specificity of patient-reported swollen joints with IA (joint swelling at physical examination as golden standard). (B) Prior probability on having IA of 41% (observed), 20% (estimated) and 2% (estimated) with corresponding post-test probabilities on having IA, if patients indicate to have ≥1 swollen joints (PPV). (C) Prior-test probability of not having IA 59% (observed), 80% (estimated) and 98% (estimated) with the corresponding post-test probability on not having IA, if patients indicate no swollen joints (NPV). IA, inflammatory arthritis; NPV, negative predictive value; PPV, positive predictive value.

The sensitivity of patient-reported joint swelling was high, 87%, indicating that the majority of patients with IA had marked swelling on the mannequin. However, the specificity was 31%, indicating that 69% of persons without IA had also done so (figure 1A). The LR+ was 1.25; the LR− 0.43. The PPV was 46%, and the NPV was 77% (figure 1B, C). Thus, the PPV increased hardly (from 41% to 46%), and the NPV somewhat increased (from 59% to 77%). Also in settings with prior-test probabilities of 20% and 2%, estimated PPVs and NPVs hardly increased (figure 1B, C).

Thus, patient-reported joint swelling had little value in distinguishing patients with and without IA, for different prior-test probabilities. Correlations identified in this population were lower than known for established RA. When evaluating ≥1 self-reported swollen and tender joints, similar results were obtained (online supplemental table S2). Together this suggests that evaluation of patient-reported swelling is less valuable for early detection of IA than for flare detection in established RA.^5 6^

Thanks to the current pandemic, telemedicine has accelerated and will continue to grow in upcoming years.^1 2^ The challenge is to continue to work in an evidence-based manner. Although inaccurate when assessed alone, patient-reported swelling may be helpful when combined with other characteristics (either clinical characteristics, such as published previously, and/or laboratory characteristics).^3 4 7 8^ Other innovative tools, for example, imaging modalities that do not require human-to-human contact, may also contribute to early identification of IA in a ‘1.5m society’ with limited access to rheumatologists.
