# Supplementary material for: Realising early recognition of arthritis in times of increased telemedicine: the value of patient-reported swollen joints
Source: Ann Rheum Dis. 2021 Jan 7;80(5):668–9. doi: 10.1136/annrheumdis-2020-219513 (PMC8053332; doi:10.1136/annrheumdis-2020-219513)
Supplement: Supplementary data [file annrheumdis-2020-219513supp001.pdf]

SUPPLEMENTARY FILES

Supplementary methods

Patients

To decrease delay in detection of early inflammatory arthritis (IA) at the level of the general practitioner (GP; referral delay), Early Arthritis *Recognition* Clinics (EA*R*C) were initiated in 2010 at the Leiden and Groningen University Medical Centers (LUMC and UMCG) in the Netherlands. The EA*R*C design of both clinics has been described previously.[1-3] In short, EA*R*Cs are clinics for patients in whom their general practitioner (GP) suspects but is unsure about the presence of IA. After GP referral, patients can visit the EA*R*C without an appointment. GPs in the region are instructed to swiftly refer any patient for whom they are unsure about the presence of IA, instead of 'wait and see' or ordering additional diagnostic tests. In particular, in accordance with Dutch national guidelines, auto-antibody testing is commonly not performed by local GPs.[4] The EA*R*C was held twice a week between 2010 and 2014 and once a week from 2014 onwards. Since the introduction of the EARC, referral delay was reduced from 8 to 2 weeks and the proportion of patients with RA that seen <12 weeks after symptom onset increased from 32 to 65%. [1] These easy-access clinics are thus characterized by their unique intermediate setting, namely in between primary and secondary care. A total of 1387 patients consecutively visited the Leiden-EA*R*C between 2012-2018 and 250 patients consecutively visited the Groningen-EA*R*C between 2012-2014. Because of the similar settings, the patients from both clinics were summed for this study. Patients included before April 2012 were not analyzed for

this study because of different mannequin formats. The study was conducted in compliance with the Helsinki declaration and was approved by the LUMC medical ethical committee.

Data collection

Patients referred to the EA*R*C completed a short questionnaire about their joint symptoms, after which they were seen by an experienced rheumatologist who performed a full 66-joint examination. If clinical apparent arthritis was present, patients were seen within 1-week at the regular outpatient clinic of the department of rheumatology for further evaluation and treatment. Patients without IA were discharged to primary care. The following questions were asked to patients who visited the EA*R*C: age, gender, date of symptom onset, date of first visit to GP and morning stiffness (duration in minutes). EA*R*C patients were asked to indicate which joints were painful and swollen (52 joints). Supplementary Figure 1 shows the mannequin completed by patients at the EARC. IA, defined as synovitis (joint swelling) confirmed by the rheumatologist at physical examination, was used as outcome. Collected data were anonymized and entered in a research database at chronological order of visiting EA*R*C. For this study the DIP joints and the metatarsal joints were excluded. DIP joints were excluded as these are the preferential locations for osteoarthritis (OA). The metatarsal joints were excluded because this specific joint is difficult to differentiate for most patients from other joints in the foot. Therefore, a total of 42 joints were assessed for self-reported joint swelling, namely MCP 1-5, PIP 1-5, MTP 1-5, Wrist, elbow, Hip, knee, ankle and shoulder.

Outcome

Patient-reported joint swelling is defined as a self-reported SJC of≥1 of the 42 included joints on the mannequin format. IA determined by the rheumatologist at physical examination (swelling of ≥1joint) was used as golden standard. Final classifying diagnoses were made during subsequent visit(s) at the regular rheumatology outpatient clinic and were beyond the scope of this study.

Patient and public involvement

Patients or the public were not involved in the design, or conduct, or reporting, or dissemination plans of our research.

Statistical analysis

We used the student –t and Mann-Whitney test to compare baseline values between patients with IA and patients without IA. The sensitivity, specificity, positive predictive value (PPV), the negative predictive value (NPV), the positive likelihood ratio (LR+) and the negative likelihood ratio (LR-) of patient-reported swelling were determined on patient-level. Predictive values depend on the prevalence of a disease in a population. Because the prevalence of IA in a 1.5-lines-setting will differ from a primary care setting, post-test probabilities of IA were estimated for two lower prior-test probabilities as example, namely 20% (an estimated probability in patients, in which GPs belief IA is likely) and 2% (~a pre-test probability with less

preselection by GPs), using likelihood ratios a nomogram. The estimated 20% was guided based on previous literature.[3] Finally a Pearson correlation between patient reported swelling and actual IA was performed on patient-level and joint-level. This was done to compare the correlation within the present patient population to the correlation that is observed in patients with established RA, as reported previously.[5] Because this data is a dichotomous categorical variable, Pearson and Spearman both had the same result. STATA software V.15 was used to analyze the data.

BMJ Publishing Group Limited (BMJ) disclaims all liability and responsibility arising from any reliance placed on this supplemental material which has been supplied by the author(s)

Supplementary Figure 1 Mannequin for patient-reported swollen joints completed by patients at the Early Arthritis Recognition Clinic

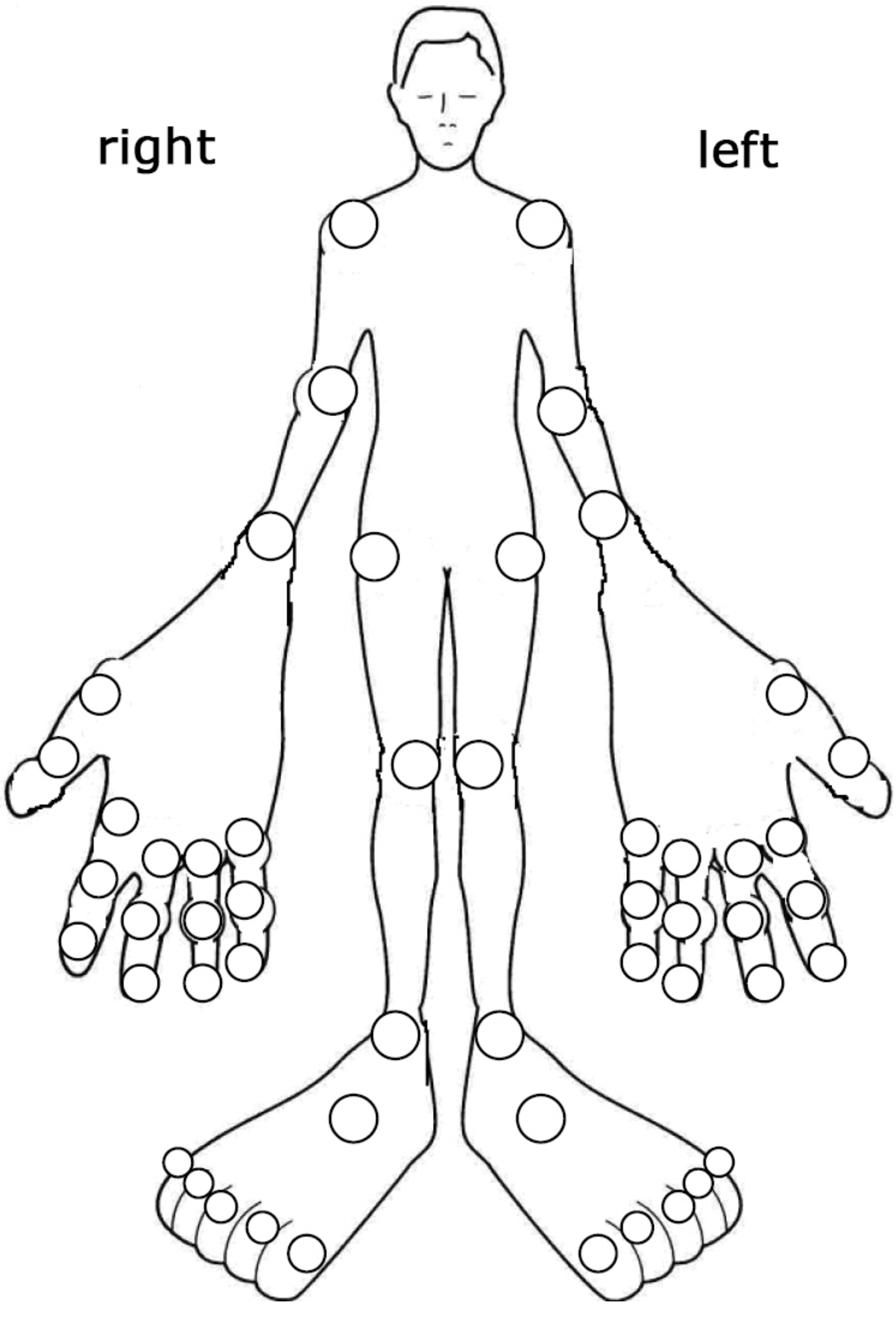

SUPPLEMENTARY RESULTS

Supplementary table 1 Baseline characteristics patients with and without inflammatory arthritis at the EA*R*C

Baseline Characteristics	All patients visiting EA*R*C (n= 1637)	Patients with Inflammatory arthritis (IA) (n= 663)	Patients without inflammatory arthritis (no-IA) (n=974)	P-value
**Female, n (%)**	1111 (68)	382 (58)	729 (75)	< 0.001
**Age, mean (SD)**	53 (17)	56 (17)	50 (16)	< 0.001
**Symptom duration in weeks, median (IQR)**	13 (4-53)	9 (3-32)	16 (5-76)	< 0.001
**Morning stiffness in minutes, median (IQR)**	10 (0-30)	10 (0-30)	10 (0-30)	0.9451
**Number of patient reported tender joints, median (IQR)**	6 (2-13)	5 (2-10)	7 (2-16)	< 0.001

Supplementary table 2 Test characteristics and predictive values of patient-reported SJC ≥1 and patient-reported TJC ≥1 with inflammatory arthritis at physical examination as reference

Sensitivity	Specificity	PPV	NPV	LR+	LR-
85% (82%-88%)	33% (30%-36%)	47% (44%-49%)	77% (73%-81%)	1.28 (1.21-1.35)	0.44 (0.36-0.54)

NOTE: Both patient-reported SJC and patient-reported TJC had to be ≥1. Clinically apparent IA of ≥1joint determined by the physician was used as reference.

Supplementary figure 2 Estimated posterior test probability in a population of patients in which GPs belief inflammatory arthritis is likely (prior test probability 20%).

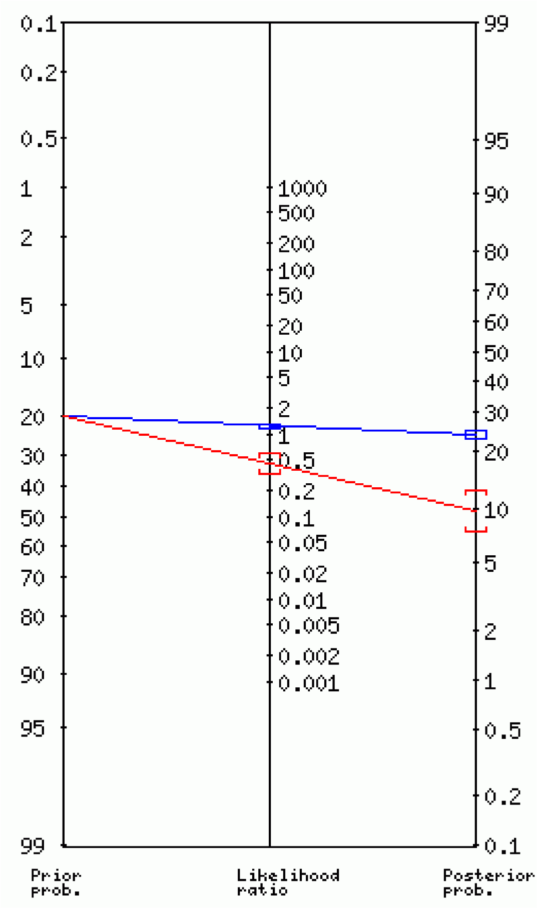

Legend: Nomogram to calculate the posterior test probability according to the estimated prior test probability and the observed likelihood ratio. In EARC population the patient-reported SJC have a sensitivity of 87% a specificity of 31% and a LR + of 1.25 and a LR- of 0.43. With a prior probability of 20% and a LR+ of 1.25 the posterior probability of having IA is 24%. With a prior probability of 20% and a LR- of 0.43 the corresponding NPV is 90%. The results from this nomogram are depicted in Fig1B,C.

Supplementary figure 3 Estimated posterior test probability in population with a pre-test probability with less preselection by GPs (prior test probability 2%).

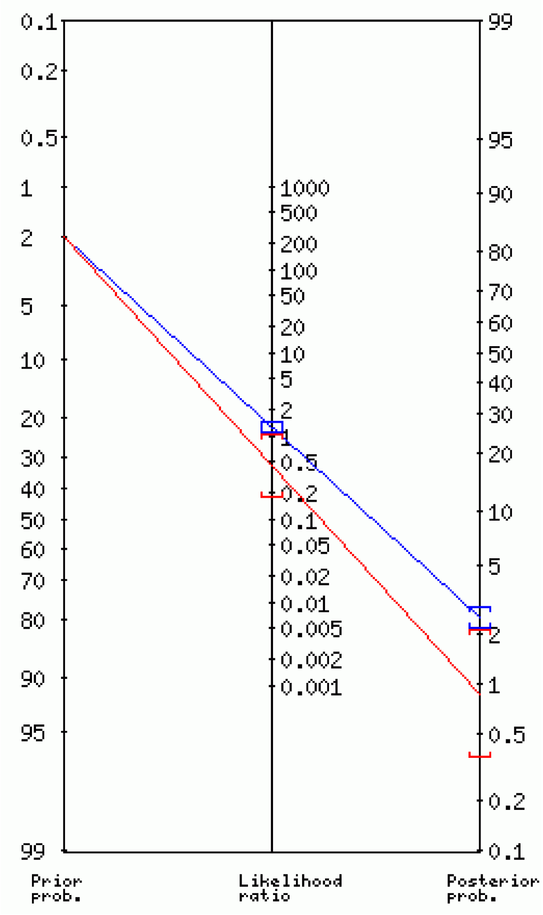

Legend: Nomogram to calculate the posterior test probability according to the estimated prior test probability and the observed likelihood ratio. In EARC population the patient-reported SJC have a sensitivity of 87% a specificity of 31% and a LR + of 1.25 and a LR- of 0.43. With a prior probability of 2% and a LR+ of 1.25 the posterior probability of having IA is still 2%. With a prior probability of 2% and a LR- of 0.43 the corresponding NPV is 99%. The results from this nomogram are depicted in Fig1B,C.

1. van Nies JA, Brouwer E, van Gaalen FA, et al. Improved early identification of arthritis: Evaluating the efficacy of early arthritis recognition clinics. *Ann Rheum Dis* 2013;72:1295-1301.
2. van Nies JA, Brouwer E, de Rooy DP, et al. Reasons for medical help-seeking behaviour of patients with recent-onset arthralgia. *Ann Rheum Dis* 2013;72:1302-1307.
3. Ten Brinck RM, van Dijk BT, van Steenbergen HW, et al. Development and validation of a clinical rule for recognition of early inflammatory arthritis. *BMJ Open* 2019;8:e023552.
4. [Internet]. Nederlands Huisartsen Ggenootshap. NHG-Standaard Artritis. Https://www.Nhg.Org/standaarden/volledig/nhg-standaard-artritis (visited on 03 nov 2020). 2017
5. Barton JL, Criswell LA, Kaiser R, et al. Systematic review and metaanalysis of patient self-report versus trained assessor joint counts in rheumatoid arthritis. *J Rheumatol* 2009;36:2635-2641.
